# Osteitis: a retrospective feasibility study comparing single-source dual-energy CT to MRI in selected patients with suspected acute gout

**DOI:** 10.1007/s00256-016-2533-1

**Published:** 2016-11-21

**Authors:** Torsten Diekhoff, Michael Scheel, Sandra Hermann, Jürgen Mews, Bernd Hamm, Kay-Geert A. Hermann

**Affiliations:** 1 Department of Radiology, Charité - Universitätsmedizin Berlin Campus Mitte, Humboldt-Universität zu Berlin, Freie Universität Berlin, Berlin, Germany; 2 Department of Radiology (CCM), Charité - Universitätsmedizin Berlin, Charitéplatz 1, 10117 Berlin, Germany; 3 Department of Rheumatology and Clinical Immunology, Charité – Universitätsmedizin Berlin Campus Mitte, Berlin, Germany; 4 Toshiba Medical Systems Europe, BV, Zilverstraat 1, 2701RP, Zoetermeer, Netherlands

**Keywords:** Dual-energy computed tomography, Bone marrow edema, Inflammation, Postprocessing

## Abstract

**Objective:**

Dual-energy computed tomography detects tophi in patients with chronic gout. However, other information that can be obtained from the same scan is not the focus of the current research, e.g., the detection of bone marrow edema (BME) using virtual bone marrow imaging (VBMI). The aim of this study was to evaluate if BME in patients with acute arthritis can be detected with VBMI using magnetic resonance imaging (MRI) as the standard of reference.

**Materials and methods:**

This retrospective study included 11 patients who underwent both MRI and dual-energy computed tomography (mean interval of 40 days). BME in MRI (standard of reference) and VBMI was judged independently by two different blinded readers. φ-correlation coefficient and Cohen’s κ were performed for statistical analysis. Approval was waived by the IRB.

**Results:**

Two patients with a final diagnosis of RA and one with septic arthritis showed osteitis on MRI and VBMI. However, in each case, there were individual bones identified with osteitis on MRI but not VBMI. Three additional patients with the final diagnosis of RA were identified correctly as negative for BME. There was a good correlation between both modalities (φ = 0.8; κ = 0.8). Inter-rater reliability was excellent for both modalities (κ = 0.9).

**Conclusions:**

We have shown that detecting osteitis using VBMI is feasible in patients with inflammatory arthritis. Further studies are needed on larger, more-targeted populations to better define the indications, accuracy, and added value of this technique.

## Introduction

Dual-energy computed tomography (DECT) is known for its ability to detect uric acid tophi in patients with chronic gouty arthritis [1–3]. Therefore, it has become a problem-solving tool in patients with unclassified arthritis and suspicion of crystal arthropathy when deemed necessary based on the clinical presentation [4]. However, DECT has many more possible applications that may be useful for clinical practice, including the virtual non-calcium technique that leads to a virtual bone marrow image (VBMI). The latter yields an image that enables detection of bone marrow edema (BME)—previously only possible with magnetic resonance imaging (MRI) [5].

VBMI is based on a three-material decomposition algorithm that allows subtraction of one material (e.g., calcium) to create an image that virtually consists only of two materials (e.g., fat and soft tissue) [5]. Therefore, the resulting images allow differentiation between adult fatty bone marrow with low attenuation in CT (∼ −100 HU) and increased water content (~0 HU) or displacement of marrow fat. Using an adjusted soft tissue window, the images display fat as black, while BME appears as gray to white.

In the last few years, only a few studies have investigated VBMI, and these have focused on trauma imaging, showing that VBMI is able to characterize the age of vertebral fractures [6, 7] and that it is comparable to MRI in displaying bone bruises on the ankle [8]. So far, only one case report published in the literature describes the use of VBMI to detect active inflammation in a patient with suspected sacroiliitis [9]. There is evidence that BME is a strong predictor of progression to bone erosions [10, 11] and cartilage damage [12] in patients with rheumatoid arthritis [13, 14], even if synovitis is a more sensitive parameter of disease activity. Recent studies were able to show that suppression of osteitis in patients with rheumatoid arthritis goes along with suppression of structural progress, e.g., erosions [15, 16]. Studies on bone remodeling suggest that osteitis in MRI is a sign of increased RANKL expression, which leads to osteoclastic bone resorption [17]. However, not all patients with suspected arthritis may be able to undergo MRI of the hands or feet due to various contraindications [18]. Finally, ultrasound has its role in visualizing extraosseous pathology such as synovitis and bone erosion, while intraosseous processes, especially BME, are hidden to the ultrasound probe [19].

We hypothesize that VBMI is able to identify osteitis. Thus, the aim of this study was to evaluate if BME in patients with acute arthritis can be detected with VBMI using magnetic resonance imaging (MRI) as a standard of reference.

## Materials and methods

We retrospectively identified all patients with suspected gout who underwent DECT in the period from September 2011 through July 2014 and additionally had an MRI of the same anatomical region. All patients had an unclear clinical presentation and suspicion of gouty arthritis that occurred either when first presenting at our hospital or later in the course of the treatment. DECT was performed to search for gouty tophi, and MRI in clinical routine to detect inflammatory changes. The report database and hospital PACS (picture archiving and communication system) were used to find those examinations. From the 24 patients who met these criteria, we selected those who had an interval of 3 months or less between DECT and MRI. Eleven patients (six men and five women aged from 45 to 81 years (mean age, 62.2 years) with an interval of 0–82 days (mean, 40 days) between examinations met our criteria and were included. Details of the study population are summarized in Table 1.

**Table 1:** Patients’ characteristics, examination, and diagnosis

No.	Age	Interval	Region	mA	DLP	EED	Diagnosis	MRI+	VBMI+
1	81	66 *	Fingers	15/90	24.1	0.019	Seropositive RA	0	0
2	45	34	Feet	25/140	38.5	0.008	Seropositive RA	8^a^	8^a^
3	74	12	Fingers	25/140	38.5	0.031	Seronegative RA	2	1
4	69	50	Wrist	15/90	24.1	0.019	CPPD	0	0
5	46	7 *	Fingers	30/170	46.6	0.037	Seronegative RA	0	0
6	69	0	Fingers	20/110	30.5	0.024	Seronegative RA	0	0
7	63	41 *	Wrist	20/110	30.5	0.024	CPPD	0	0
8	63	82	Wrist	20/110	30.5	0.024	CPPD	0	0
9	52	49	Foot	15/90	24.1	0.005	Gouty arthritis	0	0
10	59	75	Foot	25/140	38.5	0.008	Bone infarction	1	1
11	63	23 *	Ankle	15/90	24.1	0.005	Infectious arthritis	4	2

*No.* patients’ number;* Interval* interval between MRI and DECT or vice versa in days, * indicating that MRI was performed after DECT;* Region* examined body part;* mA* applied tube current for DECT examination for 135 and 80 kV,* DLP* dose-length product in mGy*cm;* EED* estimated effective dose in mSv using a conversion coefficient of 0.0008 for the upper extremities and 0.0002 for feet and ankles.* Diagnosis* final diagnosis made by the rheumatologist (*RA* rheumatoid arthritis;* CPPD* calcium pyrophosphate dihydrate crystal deposition disease);* MRI+* number of bones with BME in MRI;* VBMI+* number of bones with BME in DECT. ^a^For patient No. 2, there is one false-positive and one false-negative detection in VBMI

DECT scans were obtained on a 320-row scanner (Toshiba Aquilion ONE^TM^ and from September 2013 Toshiba Aquilion ONE Vision^TM^; Toshiba, Otawara, Japan) with 16-cm z-axis coverage without table movement using 135-kVp (high) and 80-kVp (low) tube voltage [3]. The applied tube current and the resulting radiation exposure depended on the availability of iterative reconstructions (since 2013), patients’ physique, and the examined region (see Table 1 for details). For image processing, we used proprietary virtual non-contrast software (dual-energy image view, Version 6, Toshiba, Otawara, Japan) with an adapted dual-energy gradient of 0.69 for calcium. This software is commercially available for Toshiba CT machines, e.g., Aquilion Prime or Aquilion One Vision. Object formulas for the three-material decomposition algorithm were −136/-106 (80/135 kV) for fat and 0/0 for water. A Gaussian noise reduction filter was applied. VBMI images were created in 0.5-mm slices and 5-mm averaged multiplanar reconstructions. We included MR images from different scanners and hospitals (1.5–3 Tesla) acquired using different coils (knee, hand, and flex-coil) into our analysis. An evaluation before study inclusion by reader 1 found all images to be diagnostic and not impaired by severe artifacts, e.g., due to metal implants. The MRI protocol was not the same for all patients but included at least one fat-saturated T2-weighted or fat-saturated proton density (PD) weighted sequence, which can be evaluated for the presence of BME. Slice orientation varied amongst the examinations. However, usually coronal or sagittal images were available.

Both readers (reader 1: junior radiologist with 5 years experience in image reading; reader 2: senior radiologist with 14 years experience) independently interpreted the images with an interval of at least 6 months between the reading of MR and VBMI. They used a workstation with a high-resolution monitor and OsiriX Version 5 (Pixmeo SARL, Bernex, Switzerland). The readers were blinded to clinical data, results, and images of the other imaging modality. They had access to all images of the examination including conventional multiplanar CT reconstructions (DECT) or T1 sequences (MRI). Each bone displayed in both imaging techniques was scored as BME-negative or BME-positive. A bone was counted as BME positive if both readers agreed in the presence of edema. Furthermore, the readers documented potential artifacts, e.g., due to incomplete fat saturation in MRI.

Statistical analysis was performed using GraphPad Prism (Version 6, La Jolla, CA, USA) and included the calculation of the φ correlation coefficient and Cohen’s κ to compare both imaging techniques. Cohen’s κ was also used to calculate the inter-rater reliability.

The patients’ final diagnoses were established by an expert rheumatologist based on clinical presentation, laboratory findings, and imaging results. For patient No. 11, additionally a joint aspiration was performed. The local ethics committee waived the approval of this retrospective study.

## Results

We identified 188 bones that were displayed in both MRI and VBMI. Thirty-eight bones in a total of six patients were excluded because of MRI artifacts with subsequent incomplete fat saturation, resulting in a total of 150 bones that were included in our analysis. All artifacts were caused by inhomogeneity of the magnetic field, e.g., at the tip of the toes. There were no artifacts in the VBMI reconstructions impeding image interpretation.

**Fig. 1:**
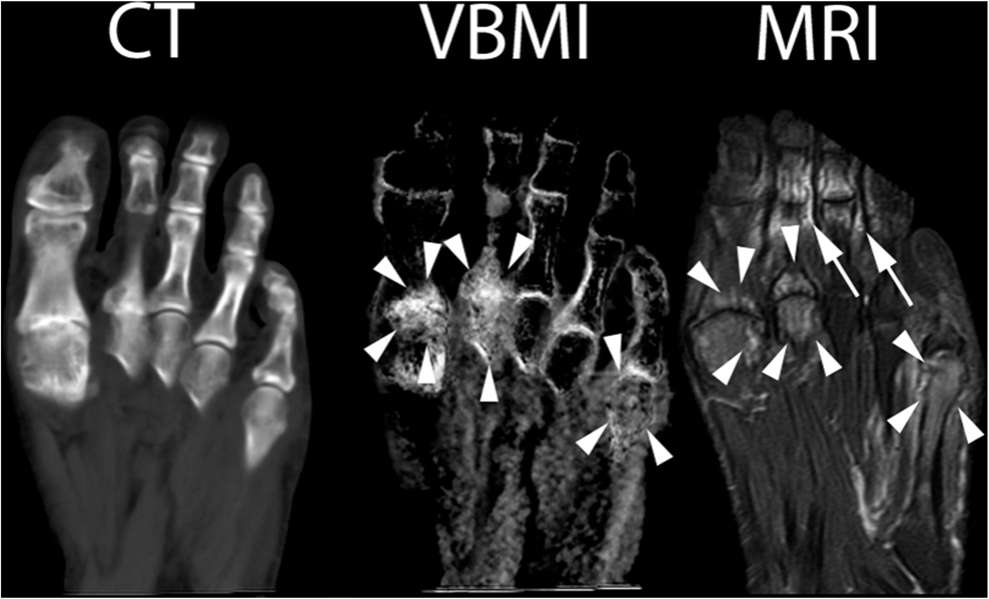
Left foot of patient No. 2 (seropositive RA). CT: normal CT image at 135 kV, VBMI: corresponding VBMI image, MRI: corresponding fat-saturated T2 sequence – multiplanar reformatted VBMI shows a good correlation to the corresponding MRI with regard to the identification of BME at the first, second, and fifth metatarsophalangeal joint (*arrowheads*). However, in MRI, there are artifacts due to incomplete fat saturation in the second and third toes (*arrows*). The corresponding CT image appearance is normal

There were five patients with a final diagnosis of rheumatoid arthritis, two with and three without osteitis in MRI, involving ten bones in total. VBMI correctly identified osteitis in both positive patients (seven true-positive, two false-negative, and one false-positive bone) and correctly showed the absence of osteitis in the three negative patients. One patient with infectious arthritis showed osteitis in MRI and VBMI, however, VBMI identified only two out of four affected bones. The bone marrow changes of another patient with bone infarction was detected by both MRI and VBMI. The other patients with the final diagnosis of crystal arthropathy showed no BME in either of the modalities. The false-negative results in VBMI occurred as discreet BME of the proximal phalanx 5 of the tarsus (patient No. 2), the intermediate phalanx 4 (patient No. 3), and the distal fibula and tibia (patient No. 11) in MRI. There was one false-positive detection in the metatarsal head (patient No. 2), as shown in Fig. 1. However, on the patients’ level, there were no false-positive or false-negative detections using VBMI. This means, each patient showing BME in MRI was detected by VBMI and each patient without BME in MRI was negative using DECT, respectively.

The φ correlation coefficient was 0.8 and Cohen’s κ was 0.8, indicating good agreement of both imaging methods. Inter-rater agreement was excellent for both modalities (MRI κ = 0.87, VBMI κ = 0.88).

**Fig. 2:**
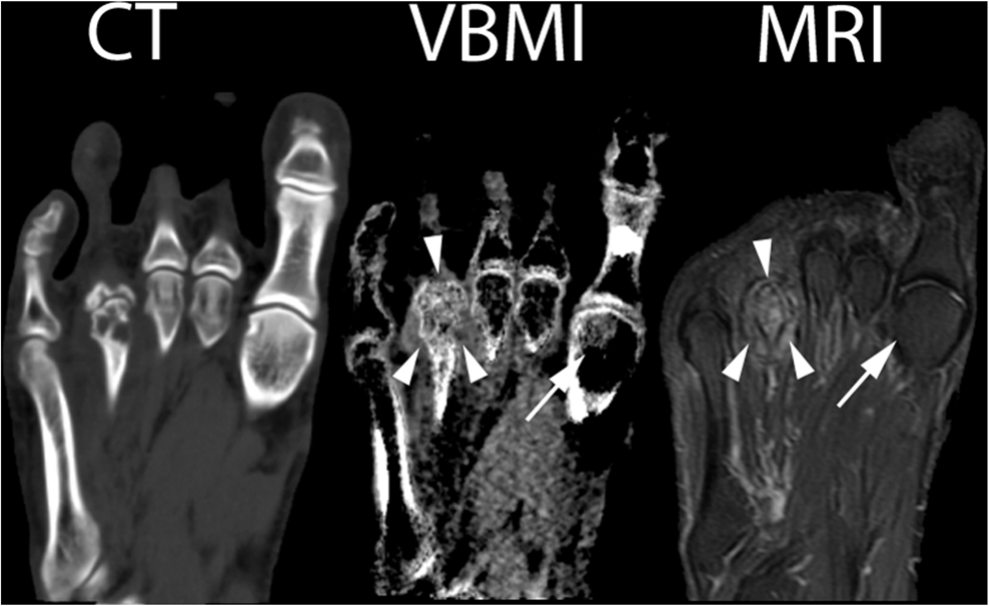
Right foot of patient No. 2 (seropositive RA). CT: normal CT image at 135 kV, VBMI: corresponding VBMI image, MRI: corresponding fat-saturated T2 sequence – VBMI displays the BME in the fourth metatarsal head (*arrowheads*) with erosions and cysts in CT. In the first metatarsal head, VBMI is false positive compared to MRI (*arrow*)

**Fig. 3:**
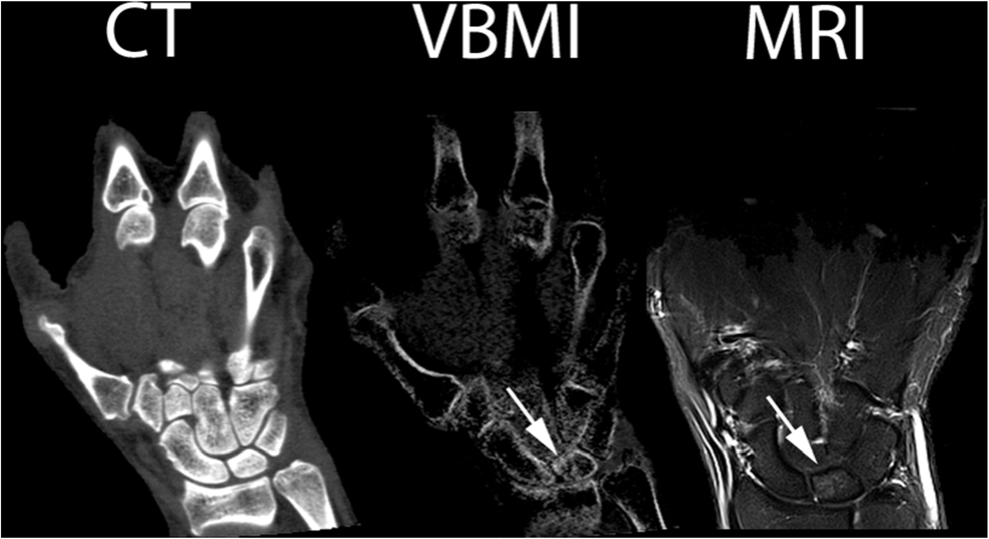
Right wrist of patient No. 7 (CPPD). CT: normal CT image at 135 kV, VBMI: corresponding VBMI image, MRI: corresponding short tau inversion recovery sequence – MRI shows a faint BME in the lunate (*arrow*) that was missed by reader 1. VBMI also displays a slight hyperdensity, which was scored negative by both readers because even normal carpal bones yield increased density in VBMI compared to long bones according to our clinical experience

**Fig. 4:**
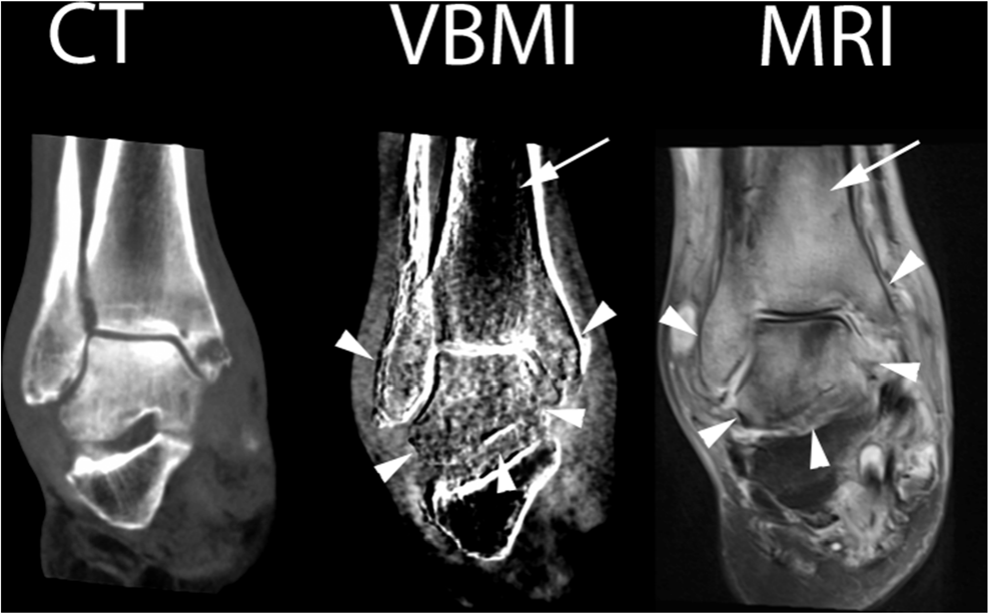
Right ankle of patient No. 11 (infectious arthritis). CT: normal CT image at 135 kV, VBMI: corresponding VBMI image, MRI: corresponding fat-saturated PD sequence – MRI shows pronounced edema in the distal tibia, fibula and calcaneus (*arrowheads*). The osteitis in the distal fibula and tibia was missed by reader 2 in VBMI. Nonetheless, VBMI depicts the edema in the distal tibia to a lesser extent compared to MRI (*arrow*)

Figures 1, 2, 3, and 4 provide examples of true-positive, false-positive, and false-negative findings.

## Discussion

In our small retrospective feasibility study, we were able to show that VBMI can detect osteitis in arthritis patients. The first results indicate a high sensitivity and specificity compared to MRI as standard of reference. Despite missing 20 % of the edema looking at single bones, all patients with BME were detected by DECT in our small cohort. The depiction of BME using VBMI may be of added value in patients undergoing a clinical DECT scan of the extremities.

DECT is an emerging technique for gaining information on material composition and physical properties of a certain tissue. In clinical practice, it is not only used to detect gouty tophi or characterize renal stones [20] but has also evolved into a tool for mapping iodine concentrations and the distribution of contrast media or for virtually subtracting contrast medium or bone of an image [21, 22]. DECT analysis of bone marrow using VBMI is another promising application for detecting BME not only in trauma or arthritis but also in malignant bone infiltration, where the technique allows estimating the permeative extension of primary bone tumor cells into normal-appearing bone marrow [23]. VBMI information is obtained by using a three-material decomposition algorithm that is able to virtually eliminate the information of calcium attenuation out of an image. In graphical terms, this can be achieved by projecting all voxels on a graph showing the attenuation of the voxel at high and low kV images parallel to the calcium gradient to a line determined by the specific object formulas of water and fat. The result is an image that is virtually based on the water and fat attenuations only and does not include the calcium information. Therefore, differences in density are related to different water and fat contents allowing to search for BME.

The fact that 38 out of 188 bones (or 20 %) in our small collective had to be excluded from analysis due to incomplete fat saturation in MRI indicates that positioning in the MRI machine plays an important role—especially for the appendicular skeleton—and is sometimes complex [24]. Incomplete fat saturation may mimic BME. Therefore, a high signal in fat-saturated MR images must be interpreted with caution. However, VBMI is independent of a homogeneous magnetic field and therefore such artifacts are considerably less frequent.

Nonetheless, VBMI uses X-rays and is therefore associated with radiation exposure. However, the investigated appendicular skeleton is far away from the radiosensitive organs such as eye lens or thyroid gland. Furthermore, the examined volume of hands and feet is considerably smaller than for example the abdomen resulting in less radiation for the same image quality. This results in a small conversion coefficient that was recently decreased for the lower extremities using new phantom measurements [25].

In our analysis, we had four false-negative results in VBMI. Mostly, these edemas detected by MRI were rather faint and small in size. Thus, they are more likely to be missed by VBMI. On the other hand, there is a rather long time interval between both imaging modalities in those cases, also in the false-positive detection on the right metatarsal bone. Therefore, the hypothesis can be made that edema might be altered due to treatment effects or worsening of the disease.

This study is a feasibility test and suffers from all limitations inherent to a retrospective design including the use of different imaging protocols and variable intervals between the examinations. However, at least for DECT, there is some evidence that radiation dose does not significantly affect the detection of BME [26]. Furthermore, our patient sample is small and heterogeneous, including complex cases and different diagnoses. This is why the first imaging test was inconclusive and both DECT and MRI were performed to secure the diagnosis. This leads to a lack of power, and statistical analysis performed in this study should therefore be treated with caution. Moreover, there was a low overall prevalence of BME in our study. This may be caused by the conservative statistical evaluation counting a bone only as positive if both readers found osteitis. We also did not collect information about the extent and severity of BME in this study. Therefore, it is left to further evaluations to determine if MRI and VBMI are comparable in depicting the degree of osteitis in patients with arthritis.

Our study gives first evidence that DECT with VBMI is capable of identifying osteitis in patients with active arthritis, so far only possible with MRI, and thus can provide additional information that may be derived from DECT scans. Furthermore, DECT may be an inexpensive and fast alternative to MRI and a useful supplement to ultrasound. Therefore, prospective data should be acquired to prove the benefit of VBMI in diagnosing arthritis in clinical practice.
